# Ethyl 4-(3-bromo-2-thien­yl)-2-oxo-6-phenyl­cyclo­hex-3-ene-1-carboxyl­ate

**DOI:** 10.1107/S1600536808002717

**Published:** 2008-02-06

**Authors:** Andreas Fischer, H. S. Yathirajan, B. V. Ashalatha, B. Narayana, B. K. Sarojini

**Affiliations:** a Inorganic Chemistry, School of Chemical Science and Engineering, Royal Institute of Technology (KTH), 100 44 Stockholm, Sweden; b Department of Studies in Chemistry, University of Mysore, Manasagangotri, Mysore 570 006, India; c Department of Chemistry, Mangalore University, Mangalagangotri 574 199, India; d Department of Chemistry, P. A. College of Engineering, Nadupadavu, Mangalore 574 153, India

## Abstract

The title compound, C_19_H_17_BrO_3_S, crystallizes with two mol­ecules in the asymmetric unit. The methyl group of one mol­ecule is disordered approximately equally over two positions. The dihedral angles between the thio­phene and phenyl groups are 68.5 (2) and 67.5 (2)° in the two mol­ecules.

## Related literature

For related structures, see Fischer *et al.* (2007*a* ▶,*b* ▶). For related literature, see: House (1972 ▶); Tabba *et al.* (1995 ▶); Dimmock *et al.* (1999 ▶).
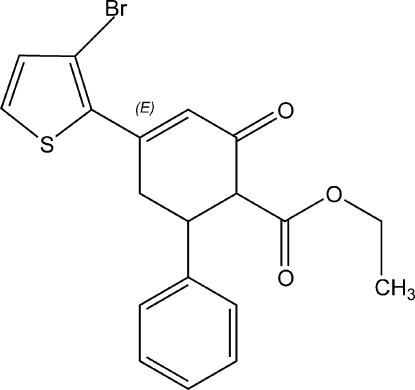


## Experimental

### 

#### Crystal data

C_19_H_17_BrO_3_S
*M* *_r_* = 405.31
Triclinic, 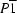

*a* = 8.8925 (8) Å
*b* = 11.713 (2) Å
*c* = 16.853 (2) Å
α = 94.317 (11)°
β = 98.436 (10)°
γ = 90.235 (13)°
*V* = 1731.3 (4) Å^3^
*Z* = 4
Mo *K*α radiation
μ = 2.50 mm^−1^
*T* = 130 K
0.30 × 0.17 × 0.05 mm

#### Data collection

Bruker Nonius KappaCCD diffractometer
Absorption correction: numerical (*HABITUS*; Herrendorf & Bärnighausen, 1997 ▶) *T*_min_ = 0.638, *T*_max_ = 0.843
40436 measured reflections
7898 independent reflections
6074 reflections with *I* > 2σ(*I*)
*R*_int_ = 0.073

#### Refinement

*R*[*F* ^2^ > 2σ(*F* ^2^)] = 0.042
*wR*(*F* ^2^) = 0.089
*S* = 1.04
7898 reflections
438 parameters
H-atom parameters constrained
Δρ_max_ = 0.84 e Å^−3^
Δρ_min_ = −0.54 e Å^−3^

### 

Data collection: *COLLECT* (Nonius, 1999 ▶); cell refinement: *DIRAX* (Duisenberg, 1992 ▶); data reduction: *EVALCCD* (Duisenberg *et al.*, 2003 ▶); program(s) used to solve structure: *SHELXS97* (Sheldrick, 2008 ▶); program(s) used to refine structure: *SHELXL97* (Sheldrick, 2008 ▶); molecular graphics: *DIAMOND* (Brandenburg, 2007 ▶); software used to prepare material for publication: *publCIF* (Westrip, 2008 ▶).

## Supplementary Material

Crystal structure: contains datablocks global, I. DOI: 10.1107/S1600536808002717/pv2059sup1.cif

Structure factors: contains datablocks I. DOI: 10.1107/S1600536808002717/pv2059Isup2.hkl

Additional supplementary materials:  crystallographic information; 3D view; checkCIF report

## Figures and Tables

**Fig. 1.**
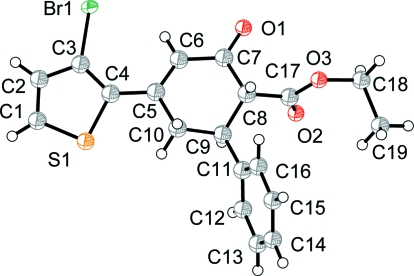
The structure of molecule 1 in the asymmetric unit; displacement ellipsoids have been plotted at the 50% probability level. Only one of the conformational isomers is shown.

**Fig. 2.**
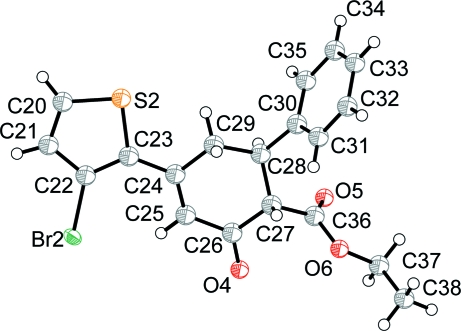
The structure of molecule 2 in the asymmetric unit; displacement ellipsoids have been plotted at the 50% probability level.

## supplementary crystallographic information

### Comment

Chalcones and the corresponding heterocyclic analogs are valuable intermediates in organic synthesis and exhibit a multitude of biological activities (Dimmock *et al.*, 1999). An important feature of chalcones and their heteroanalogs is the ability to act as activated unsaturated systems in conjugated addition reactions of carbanions in the presence of basic catalysts (House, 1972). This type of reaction may be exploited with the goal of obtaining highly functionalized cyclohexene derivatives (Tabba *et al.*, 1995). In view of the importance of these derivatives and continuing our efforts in this field (Fischer *et al.*, 2007*a*; 2007*b*), a new derivative, the title compound, (I), has been prepared and its crystal structure is reported in this paper.

The structure of (I) contains two molecules in an asymmetric unit (Figs. 1 and 2). A methyl C-atom of methoxy group in the molecule 1, presented in Fig. 1 is disordered over two sites C19 and C19'. The geometry in the two molecules is unexceptional. The crystal packing is stabilized by van der Waals forces. The dihedral angles between the thiophene groups and phenyl groups in the two molecules are 68.5 (2) and 67.5 (2)°.

### Experimental

(2*E*)-1-(3-Bromo-2-thienyl)-3-phenylprop-2-en-1-one (1.5 g, 5 mmol) and ethyl acetoacetate (0.65 g, 5 mmol) were refluxed for 2 hr in 10–15 ml e thanol in the presence of 0.8 ml 10% NaOH. The reaction mixture was cooled to room temperature and the reaction mass was filtered and recrystallized using methanol. Crystals were grown from acetone (m.p. 399–400 K).

### Refinement

Hydrogen atoms were placed at calculated positions with C—H distances: 0.95, 0.98 and 0.99 Å for aromatic, methyl and methylene groups, respectively, and were included in the refinements in riding mode with *U*_iso_ = 1.2 and 1.5 time *U*_eq_ of the carrier atoms for non-methyl and methyl groups, respectively. A methyl C-atom of methoxy group in molecule 1 was disordered over two positions C19 and C19' with site occupation factors of 0.513 (6) and 0.487 (6), respectively.

### Crystal data

**Table:**

C_19_H_17_BrO_3_S	*Z* = 4
*M**_r_* = 405.32	*F*_000_ = 824
Triclinic, *P*1	*D*_x_ = 1.555 Mg m^−^^3^
Hall symbol: -P 1	Mo *K*α radiation λ = 0.71073 Å
*a* = 8.8925 (8) Å	Cell parameters from 44 reflections
*b* = 11.713 (2) Å	θ = 6.3–19.4º
*c* = 16.853 (2) Å	µ = 2.50 mm^−^^1^
α = 94.317 (11)º	*T* = 130 K
β = 98.436 (10)º	Plate, colourless
γ = 90.235 (13)º	0.30 × 0.17 × 0.05 mm
*V* = 1731.3 (4) Å^3^	

### Data collection

**Table:**

Bruker Nonius KappaCCD diffractometer	6074 reflections with *I* > 2σ(*I*)
Radiation source: fine-focus sealed tube	*R*_int_ = 0.073
φ and ω scans	θ_max_ = 27.5º
Absorption correction: numerical(HABITUS; Herrendorf & Bärnighausen, 1997)	θ_min_ = 4.5º
*T*_min_ = 0.638, *T*_max_ = 0.843	*h* = −11→11
40436 measured reflections	*k* = −15→15
7898 independent reflections	*l* = −21→21

### Refinement

**Table:**

Refinement on *F*^2^	Secondary atom site location: difference Fourier map
Least-squares matrix: full	H-atom parameters constrained
*R*[*F*^2^ > 2σ(*F*^2^)] = 0.042	*w* = 1/[σ^2^(*F*_o_^2^) + (0.0305*P*)^2^ + 2.19*P*] where *P* = (*F*_o_^2^ + 2*F*_c_^2^)/3
*wR*(*F*^2^) = 0.090	(Δ/σ)_max_ = 0.001
*S* = 1.04	Δρ_max_ = 0.84 e Å^−^^3^
7898 reflections	Δρ_min_ = −0.54 e Å^−^^3^
438 parameters	Extinction correction: none
Primary atom site location: structure-invariant direct methods	

### Special details

**Table:**

Geometry. All e.s.d.'s (except the e.s.d. in the dihedral angle between two l.s. planes) are estimated using the full covariance matrix. The cell e.s.d.'s are taken into account individually in the estimation of e.s.d.'s in distances, angles and torsion angles; correlations between e.s.d.'s in cell parameters are only used when they are defined by crystal symmetry. An approximate (isotropic) treatment of cell e.s.d.'s is used for estimating e.s.d.'s involving l.s. planes.
Refinement. Refinement of *F*^2^ against ALL reflections. The weighted *R*-factor *wR* and goodness of fit *S* are based on *F*^2^, conventional *R*-factors *R* are based on *F*, with *F* set to zero for negative *F*^2^. The threshold expression of *F*^2^ > σ(*F*^2^) is used only for calculating *R*-factors(gt) *etc*. and is not relevant to the choice of reflections for refinement. *R*-factors based on *F*^2^ are statistically about twice as large as those based on *F*, and *R*- factors based on ALL data will be even larger.

### Fractional atomic coordinates and isotropic or equivalent isotropic displacement parameters (Å^2^)

**Table:**

	*x*	*y*	*z*	*U*_iso_*/*U*_eq_	Occ. (<1)
Br1	0.99977 (4)	0.72535 (2)	0.421420 (19)	0.03009 (9)	
S1	0.66855 (8)	0.46246 (6)	0.45787 (4)	0.02458 (16)	
O1	1.1493 (2)	0.73022 (18)	0.71856 (13)	0.0311 (5)	
O2	1.1039 (3)	0.52832 (19)	0.83761 (14)	0.0368 (5)	
O3	1.0313 (3)	0.69989 (19)	0.88526 (14)	0.0408 (6)	
C1	0.6729 (3)	0.4794 (3)	0.35854 (18)	0.0263 (6)	
C2	0.7753 (3)	0.5611 (2)	0.34871 (18)	0.0236 (6)	
C3	0.8510 (3)	0.6105 (2)	0.42277 (17)	0.0212 (6)	
C4	0.8073 (3)	0.5679 (2)	0.49033 (17)	0.0203 (6)	
C5	0.8539 (3)	0.5935 (2)	0.57634 (17)	0.0197 (6)	
C6	0.9826 (3)	0.6524 (2)	0.60774 (18)	0.0214 (6)	
C7	1.0306 (3)	0.6775 (2)	0.69339 (18)	0.0225 (6)	
C8	0.9305 (3)	0.6373 (2)	0.75173 (17)	0.0213 (6)	
C9	0.8343 (3)	0.5321 (2)	0.71617 (17)	0.0208 (6)	
C10	0.7514 (3)	0.5522 (2)	0.63266 (16)	0.0206 (6)	
C11	0.7227 (3)	0.4959 (2)	0.76997 (17)	0.0209 (6)	
C12	0.7020 (3)	0.3799 (2)	0.77736 (19)	0.0285 (7)	
C13	0.5923 (4)	0.3416 (3)	0.8206 (2)	0.0361 (8)	
C14	0.5035 (4)	0.4191 (3)	0.8572 (2)	0.0374 (8)	
C15	0.5246 (4)	0.5351 (3)	0.8508 (2)	0.0350 (7)	
C16	0.6338 (3)	0.5729 (2)	0.80828 (18)	0.0263 (6)	
C17	1.0313 (3)	0.6139 (2)	0.82876 (18)	0.0251 (6)	
C18	1.1320 (5)	0.6868 (4)	0.9614 (2)	0.0555 (11)	
C19	1.2037 (10)	0.8086 (8)	0.9813 (5)	0.0623 (17)	0.513 (6)
C19'	1.0611 (10)	0.5946 (9)	1.0106 (5)	0.0623 (17)	0.487 (6)
Br2	0.50916 (3)	0.75741 (2)	0.418423 (18)	0.02812 (9)	
S2	0.16982 (8)	1.01911 (6)	0.45701 (4)	0.02397 (16)	
O4	0.6550 (2)	0.81534 (18)	0.71690 (13)	0.0306 (5)	
O5	0.6026 (2)	1.03913 (17)	0.84067 (13)	0.0324 (5)	
O6	0.5409 (2)	0.86917 (17)	0.88122 (12)	0.0286 (5)	
C20	0.1748 (3)	0.9822 (3)	0.35764 (18)	0.0261 (6)	
C21	0.2799 (3)	0.9014 (2)	0.34699 (18)	0.0241 (6)	
C22	0.3574 (3)	0.8687 (2)	0.42091 (17)	0.0201 (6)	
C23	0.3116 (3)	0.9236 (2)	0.48873 (17)	0.0195 (6)	
C24	0.3592 (3)	0.9170 (2)	0.57463 (17)	0.0186 (6)	
C25	0.4871 (3)	0.8656 (2)	0.60608 (17)	0.0215 (6)	
C26	0.5353 (3)	0.8602 (2)	0.69166 (18)	0.0217 (6)	
C27	0.4338 (3)	0.9107 (2)	0.75024 (16)	0.0190 (6)	
C28	0.3404 (3)	1.0092 (2)	0.71490 (16)	0.0191 (6)	
C29	0.2568 (3)	0.9709 (2)	0.63110 (16)	0.0202 (6)	
C30	0.2268 (3)	1.0582 (2)	0.76721 (17)	0.0218 (6)	
C31	0.1404 (3)	0.9897 (3)	0.80725 (19)	0.0290 (7)	
C32	0.0259 (4)	1.0375 (3)	0.8464 (2)	0.0387 (8)	
C33	−0.0024 (4)	1.1527 (3)	0.8463 (2)	0.0412 (9)	
C34	0.0858 (4)	1.2217 (3)	0.8088 (2)	0.0396 (8)	
C35	0.1999 (4)	1.1751 (3)	0.76958 (18)	0.0293 (7)	
C36	0.5346 (3)	0.9491 (2)	0.82818 (18)	0.0225 (6)	
C37	0.6474 (4)	0.8921 (3)	0.9551 (2)	0.0422 (9)	
C38	0.6280 (6)	0.7991 (4)	1.0081 (3)	0.0655 (13)	
H1	0.6111	0.4369	0.3155	0.032*	
H2	0.7940	0.5825	0.2977	0.028*	
H6	1.0451	0.6788	0.5716	0.026*	
H8	0.8608	0.7005	0.7640	0.026*	
H9	0.9050	0.4673	0.7097	0.025*	
H10A	0.7006	0.4800	0.6083	0.025*	
H10B	0.6718	0.6095	0.6383	0.025*	
H12	0.7632	0.3260	0.7526	0.034*	
H13	0.5787	0.2620	0.8250	0.043*	
H14	0.4284	0.3933	0.8865	0.045*	
H15	0.4635	0.5888	0.8758	0.042*	
H16	0.6484	0.6527	0.8052	0.032*	
H18A	1.2102	0.6289	0.9544	0.067*	0.513 (6)
H18B	1.0740	0.6651	1.0040	0.067*	0.513 (6)
H19A	1.2746	0.8103	1.0317	0.093*	0.513 (6)
H19B	1.1235	0.8640	0.9872	0.093*	0.513 (6)
H19C	1.2581	0.8283	0.9376	0.093*	0.513 (6)
H18C	1.1448	0.7611	0.9939	0.067*	0.487 (6)
H18D	1.2330	0.6618	0.9500	0.067*	0.487 (6)
H19D	1.1287	0.5856	1.0610	0.093*	0.487 (6)
H19E	1.0488	0.5211	0.9784	0.093*	0.487 (6)
H19F	0.9619	0.6203	1.0226	0.093*	0.487 (6)
H20	0.1117	1.0143	0.3149	0.031*	
H21	0.2992	0.8704	0.2958	0.029*	
H25	0.5490	0.8310	0.5700	0.026*	
H27	0.3627	0.8495	0.7611	0.023*	
H28	0.4128	1.0723	0.7087	0.023*	
H29A	0.1761	0.9149	0.6366	0.024*	
H29B	0.2074	1.0379	0.6068	0.024*	
H31	0.1593	0.9102	0.8080	0.035*	
H32	−0.0334	0.9900	0.8735	0.046*	
H33	−0.0823	1.1843	0.8720	0.049*	
H34	0.0684	1.3016	0.8097	0.048*	
H35	0.2606	1.2236	0.7440	0.035*	
H37A	0.6266	0.9671	0.9820	0.051*	
H37B	0.7527	0.8938	0.9429	0.051*	
H38A	0.5228	0.7968	1.0185	0.098*	
H38B	0.6961	0.8135	1.0592	0.098*	
H38C	0.6524	0.7257	0.9816	0.098*	

### Atomic displacement parameters (Å^2^)

**Table:**

	*U*^11^	*U*^22^	*U*^33^	*U*^12^	*U*^13^	*U*^23^
Br1	0.03887 (18)	0.02276 (15)	0.02951 (18)	−0.00756 (12)	0.00681 (13)	0.00493 (12)
S1	0.0186 (3)	0.0318 (4)	0.0222 (4)	−0.0066 (3)	0.0026 (3)	−0.0035 (3)
O1	0.0262 (11)	0.0335 (12)	0.0313 (12)	−0.0141 (9)	0.0019 (9)	−0.0072 (9)
O2	0.0390 (13)	0.0326 (12)	0.0364 (14)	0.0065 (10)	−0.0033 (10)	0.0034 (10)
O3	0.0517 (14)	0.0370 (13)	0.0294 (13)	−0.0003 (11)	−0.0008 (11)	−0.0113 (10)
C1	0.0234 (15)	0.0328 (16)	0.0213 (15)	0.0020 (12)	0.0015 (12)	−0.0043 (12)
C2	0.0249 (14)	0.0255 (14)	0.0206 (15)	0.0069 (12)	0.0031 (12)	0.0042 (12)
C3	0.0198 (13)	0.0177 (13)	0.0267 (16)	0.0039 (11)	0.0057 (11)	0.0007 (11)
C4	0.0153 (13)	0.0179 (13)	0.0273 (16)	0.0020 (10)	0.0036 (11)	−0.0022 (11)
C5	0.0190 (13)	0.0163 (13)	0.0241 (15)	0.0027 (10)	0.0046 (11)	0.0010 (11)
C6	0.0168 (13)	0.0217 (14)	0.0263 (16)	−0.0031 (11)	0.0059 (11)	0.0003 (11)
C7	0.0200 (14)	0.0172 (13)	0.0297 (16)	−0.0018 (11)	0.0040 (12)	−0.0026 (11)
C8	0.0195 (13)	0.0202 (13)	0.0231 (15)	−0.0019 (11)	0.0009 (11)	−0.0009 (11)
C9	0.0229 (14)	0.0175 (13)	0.0214 (15)	−0.0033 (11)	0.0027 (11)	−0.0020 (11)
C10	0.0184 (13)	0.0236 (14)	0.0191 (15)	−0.0044 (11)	0.0020 (11)	−0.0011 (11)
C11	0.0217 (14)	0.0219 (14)	0.0180 (14)	−0.0040 (11)	−0.0007 (11)	0.0012 (11)
C12	0.0357 (17)	0.0211 (14)	0.0285 (17)	−0.0019 (13)	0.0047 (13)	0.0012 (12)
C13	0.048 (2)	0.0258 (16)	0.0357 (19)	−0.0106 (15)	0.0062 (16)	0.0073 (14)
C14	0.0434 (19)	0.0421 (19)	0.0288 (18)	−0.0178 (16)	0.0123 (15)	0.0051 (15)
C15	0.0362 (18)	0.0385 (18)	0.0319 (19)	−0.0043 (15)	0.0127 (14)	−0.0016 (14)
C16	0.0298 (16)	0.0204 (14)	0.0287 (17)	−0.0031 (12)	0.0044 (13)	0.0023 (12)
C17	0.0242 (15)	0.0249 (15)	0.0253 (16)	−0.0048 (12)	0.0034 (12)	−0.0022 (12)
C18	0.061 (3)	0.070 (3)	0.028 (2)	−0.002 (2)	−0.0077 (18)	−0.0136 (19)
C19	0.061 (4)	0.093 (5)	0.031 (3)	−0.031 (3)	−0.003 (3)	0.013 (3)
C18'	0.061 (3)	0.070 (3)	0.028 (2)	−0.002 (2)	−0.0077 (18)	−0.0136 (19)
C19'	0.061 (4)	0.093 (5)	0.031 (3)	−0.031 (3)	−0.003 (3)	0.013 (3)
Br2	0.03497 (17)	0.02113 (15)	0.02934 (17)	0.00523 (12)	0.00939 (13)	−0.00052 (12)
S2	0.0190 (3)	0.0310 (4)	0.0221 (4)	0.0037 (3)	0.0031 (3)	0.0031 (3)
O4	0.0284 (11)	0.0332 (11)	0.0313 (12)	0.0125 (9)	0.0048 (9)	0.0088 (9)
O5	0.0354 (12)	0.0249 (11)	0.0342 (13)	−0.0089 (9)	−0.0044 (10)	0.0040 (9)
O6	0.0309 (11)	0.0289 (11)	0.0246 (12)	−0.0072 (9)	−0.0035 (9)	0.0083 (9)
C20	0.0233 (15)	0.0327 (16)	0.0220 (16)	−0.0025 (12)	0.0013 (12)	0.0041 (12)
C21	0.0248 (14)	0.0259 (14)	0.0216 (15)	−0.0086 (12)	0.0060 (12)	−0.0024 (12)
C22	0.0169 (13)	0.0174 (13)	0.0259 (16)	−0.0049 (10)	0.0043 (11)	−0.0005 (11)
C23	0.0162 (13)	0.0165 (13)	0.0263 (15)	−0.0050 (10)	0.0049 (11)	0.0023 (11)
C24	0.0186 (13)	0.0147 (12)	0.0232 (15)	−0.0047 (10)	0.0063 (11)	0.0004 (11)
C25	0.0214 (14)	0.0184 (13)	0.0262 (16)	−0.0001 (11)	0.0089 (11)	0.0010 (11)
C26	0.0199 (14)	0.0160 (13)	0.0301 (16)	−0.0012 (11)	0.0049 (12)	0.0050 (11)
C27	0.0201 (13)	0.0163 (13)	0.0211 (15)	−0.0036 (10)	0.0034 (11)	0.0036 (11)
C28	0.0183 (13)	0.0182 (13)	0.0204 (15)	−0.0007 (10)	0.0022 (11)	0.0007 (11)
C29	0.0187 (13)	0.0214 (13)	0.0215 (15)	0.0018 (11)	0.0042 (11)	0.0052 (11)
C30	0.0195 (13)	0.0277 (14)	0.0161 (14)	0.0044 (11)	−0.0021 (11)	−0.0022 (11)
C31	0.0287 (16)	0.0287 (16)	0.0294 (17)	−0.0021 (13)	0.0065 (13)	−0.0024 (13)
C32	0.0329 (18)	0.056 (2)	0.0289 (19)	−0.0015 (16)	0.0122 (14)	−0.0029 (16)
C33	0.0376 (19)	0.059 (2)	0.0263 (18)	0.0207 (17)	0.0074 (15)	−0.0065 (16)
C34	0.054 (2)	0.0369 (18)	0.0260 (18)	0.0212 (16)	0.0003 (16)	−0.0033 (14)
C35	0.0348 (17)	0.0294 (16)	0.0230 (16)	0.0075 (13)	0.0020 (13)	0.0019 (13)
C36	0.0186 (13)	0.0240 (15)	0.0252 (16)	0.0005 (11)	0.0033 (11)	0.0033 (12)
C37	0.048 (2)	0.044 (2)	0.0289 (19)	−0.0130 (16)	−0.0134 (15)	0.0074 (15)
C38	0.095 (3)	0.053 (2)	0.040 (2)	−0.020 (2)	−0.027 (2)	0.0200 (19)

### Geometric parameters (Å, °)

**Table:**

Br1—C3	1.888 (3)	C30—C31	1.385 (4)
S1—C1	1.706 (3)	C30—C35	1.392 (4)
S1—C4	1.743 (3)	C31—C32	1.393 (4)
O1—C7	1.225 (3)	C32—C33	1.376 (5)
O2—C17	1.201 (3)	C33—C34	1.374 (5)
O3—C17	1.333 (4)	C34—C35	1.386 (4)
O3—C18	1.474 (4)	C37—C38	1.486 (5)
C1—C2	1.355 (4)	C1—H1	0.9500
C2—C3	1.410 (4)	C2—H2	0.9500
C3—C4	1.382 (4)	C6—H6	0.9500
C4—C5	1.455 (4)	C8—H8	1.0000
C5—C6	1.353 (4)	C9—H9	1.0000
C5—C10	1.512 (4)	C10—H10A	0.9900
C6—C7	1.451 (4)	C10—H10B	0.9900
C7—C8	1.519 (4)	C12—H12	0.9500
C8—C17	1.511 (4)	C13—H13	0.9500
C8—C9	1.530 (4)	C14—H14	0.9500
C9—C11	1.520 (4)	C15—H15	0.9500
C9—C10	1.526 (4)	C16—H16	0.9500
C11—C12	1.389 (4)	C18—H18A	0.9900
C11—C16	1.390 (4)	C18—H18B	0.9900
C12—C13	1.394 (4)	C19—H19A	0.9800
C13—C14	1.378 (5)	C19—H19B	0.9800
C14—C15	1.386 (5)	C19—H19C	0.9800
C15—C16	1.380 (4)	C19'—H19D	0.9800
C18—C19	1.554 (9)	C19'—H19E	0.9800
Br2—C22	1.883 (3)	C19'—H19F	0.9800
S2—C20	1.704 (3)	C20—H20	0.9500
S2—C23	1.741 (3)	C21—H21	0.9500
O4—C26	1.223 (3)	C25—H25	0.9500
O5—C36	1.203 (3)	C27—H27	1.0000
O6—C36	1.339 (3)	C28—H28	1.0000
O6—C37	1.457 (4)	C29—H29A	0.9900
C20—C21	1.358 (4)	C29—H29B	0.9900
C21—C22	1.414 (4)	C31—H31	0.9500
C22—C23	1.384 (4)	C32—H32	0.9500
C23—C24	1.455 (4)	C33—H33	0.9500
C24—C25	1.351 (4)	C34—H34	0.9500
C24—C29	1.520 (4)	C35—H35	0.9500
C25—C26	1.449 (4)	C37—H37A	0.9900
C26—C27	1.524 (4)	C37—H37B	0.9900
C27—C36	1.515 (4)	C38—H38A	0.9800
C27—C28	1.534 (4)	C38—H38B	0.9800
C28—C30	1.525 (4)	C38—H38C	0.9800
C28—C29	1.530 (4)		
			
C1—S1—C4	93.37 (14)	C5—C6—H6	118.1
C17—O3—C18	115.6 (3)	C7—C6—H6	118.1
C2—C1—S1	111.6 (2)	C17—C8—H8	108.4
C1—C2—C3	112.3 (3)	C7—C8—H8	108.4
C4—C3—C2	115.1 (2)	C9—C8—H8	108.4
C4—C3—Br1	126.4 (2)	C11—C9—H9	107.5
C2—C3—Br1	118.5 (2)	C10—C9—H9	107.5
C3—C4—C5	133.4 (2)	C8—C9—H9	107.5
C3—C4—S1	107.7 (2)	C5—C10—H10A	108.8
C5—C4—S1	118.9 (2)	C9—C10—H10A	108.8
C6—C5—C4	123.3 (3)	C5—C10—H10B	108.8
C6—C5—C10	119.0 (3)	C9—C10—H10B	108.8
C4—C5—C10	117.7 (2)	H10A—C10—H10B	107.7
C5—C6—C7	123.8 (3)	C11—C12—H12	119.6
O1—C7—C6	121.0 (3)	C13—C12—H12	119.6
O1—C7—C8	120.3 (3)	C14—C13—H13	120.0
C6—C7—C8	118.6 (2)	C12—C13—H13	120.0
C17—C8—C7	108.0 (2)	C13—C14—H14	120.2
C17—C8—C9	111.7 (2)	C15—C14—H14	120.2
C7—C8—C9	111.9 (2)	C16—C15—H15	119.8
C11—C9—C10	110.4 (2)	C14—C15—H15	119.8
C11—C9—C8	113.2 (2)	C15—C16—H16	119.6
C10—C9—C8	110.5 (2)	C11—C16—H16	119.6
C5—C10—C9	113.8 (2)	O3—C18—H18A	111.4
C12—C11—C16	118.4 (3)	C19—C18—H18A	111.4
C12—C11—C9	118.4 (3)	O3—C18—H18B	111.4
C16—C11—C9	123.0 (2)	C19—C18—H18B	111.4
C11—C12—C13	120.7 (3)	H18A—C18—H18B	109.2
C14—C13—C12	120.0 (3)	C18—C19—H19A	109.5
C13—C14—C15	119.6 (3)	C18—C19—H19B	109.5
C16—C15—C14	120.4 (3)	H19A—C19—H19B	109.5
C15—C16—C11	120.8 (3)	C18—C19—H19C	109.5
O2—C17—O3	124.2 (3)	H19A—C19—H19C	109.5
O2—C17—C8	123.9 (3)	H19B—C19—H19C	109.5
O3—C17—C8	111.8 (3)	H19D—C19'—H19E	109.5
O3—C18—C19	102.0 (4)	H19D—C19'—H19F	109.5
C20—S2—C23	93.22 (14)	H19E—C19'—H19F	109.5
C36—O6—C37	116.0 (2)	C21—C20—H20	124.1
C21—C20—S2	111.8 (2)	S2—C20—H20	124.1
C20—C21—C22	112.2 (3)	C20—C21—H21	123.9
C23—C22—C21	114.8 (3)	C22—C21—H21	123.9
C23—C22—Br2	126.7 (2)	C24—C25—H25	118.1
C21—C22—Br2	118.4 (2)	C26—C25—H25	118.1
C22—C23—C24	133.3 (3)	C36—C27—H27	108.7
C22—C23—S2	107.9 (2)	C26—C27—H27	108.7
C24—C23—S2	118.8 (2)	C28—C27—H27	108.7
C25—C24—C23	123.8 (2)	C30—C28—H28	107.6
C25—C24—C29	119.0 (3)	C29—C28—H28	107.6
C23—C24—C29	117.1 (2)	C27—C28—H28	107.6
C24—C25—C26	123.8 (3)	C24—C29—H29A	108.9
O4—C26—C25	120.9 (3)	C28—C29—H29A	108.9
O4—C26—C27	120.3 (3)	C24—C29—H29B	108.9
C25—C26—C27	118.8 (2)	C28—C29—H29B	108.9
C36—C27—C26	107.7 (2)	H29A—C29—H29B	107.7
C36—C27—C28	112.0 (2)	C30—C31—H31	120.0
C26—C27—C28	111.1 (2)	C32—C31—H31	120.0
C30—C28—C29	109.2 (2)	C33—C32—H32	119.6
C30—C28—C27	114.3 (2)	C31—C32—H32	119.6
C29—C28—C27	110.2 (2)	C34—C33—H33	120.2
C24—C29—C28	113.6 (2)	C32—C33—H33	120.2
C31—C30—C35	118.6 (3)	C33—C34—H34	119.9
C31—C30—C28	122.5 (3)	C35—C34—H34	119.9
C35—C30—C28	118.7 (3)	C34—C35—H35	119.6
C30—C31—C32	120.0 (3)	C30—C35—H35	119.6
C33—C32—C31	120.7 (3)	O6—C37—H37A	110.2
C34—C33—C32	119.6 (3)	C38—C37—H37A	110.2
C33—C34—C35	120.2 (3)	O6—C37—H37B	110.2
C34—C35—C30	120.8 (3)	C38—C37—H37B	110.2
O5—C36—O6	124.0 (3)	H37A—C37—H37B	108.5
O5—C36—C27	124.5 (3)	C37—C38—H38A	109.5
O6—C36—C27	111.4 (2)	C37—C38—H38B	109.5
O6—C37—C38	107.5 (3)	H38A—C38—H38B	109.5
C2—C1—H1	124.2	C37—C38—H38C	109.5
S1—C1—H1	124.2	H38A—C38—H38C	109.5
C1—C2—H2	123.9	H38B—C38—H38C	109.5
C3—C2—H2	123.9		
